# Alzheimer and vascular dementia in the elderly patients

**DOI:** 10.12669/pjms.325.10792

**Published:** 2016

**Authors:** Naresh Kumar Seetlani, Narindar Kumar, Khalid Imran, Asif Ali, Nadia Shams, Taha Sheikh

**Affiliations:** 1 Naresh Kumar Seetlani, MBBS, FCPS. Assistant Professor, Dow Medical College, Dow University of Health Sciences, Karachi, Pakistan; 2 Narindar Kumar, MBBS, FCPS. Resident, Ziauddin Medical University, Karachi, Pakistan; 3 Khalid Imran, MBBS, FCPS. Professor of Medicine, Dow International Medical College, Karachi, Pakistan; 4 Asif Ali, MBBS, FCPS. Registrar, Civil Hospital Karachi, Pakistan; 5 Nadia Shams, MBBS, FCPS. Assistant Professor of Medicine, RIHS Islamabad, Pakistan; 6 Taha Sheikh, MBBS. Final year Medical Student, Dow Medical College, Dow University of Health Sciences, Karachi, Pakistan

**Keywords:** Alzheimer’s disease, Dementia, Vascular dementia

## Abstract

**Objectives:**

To find out the frequency of Alzheimer’s and Vascular dementia in the elderly patients.

**Methods:**

This cross sectional descriptive study was conducted in Department of Medicine, Ziauddin Hospital Karachi from 1^st^ October 2013 to 31^st^ March 2014. Patients with symptoms of dementia for more than 6 months duration, and Mini Mental State Examination score <24 were included in this study. Patients who fell in category of dementia were assessed for duration of symptoms. Patients underwent CT scan of brain. Patients with generalized atrophy of brain on CT scanning of brain were labeled as Alzheimer’s dementia, while patients with ischemic or hemorrhagic stroke on CT scan of brain were labeled as vascular dementia.

**Results:**

Four hundred twenty two patients were included in this study. There were 232 (54.98 %) male and 190 (45.02 %) were female. The mean age ± SD of the patients was 72.58±5.34 years (95% CI: 72.07 to 73.09), similarly average duration of symptoms was 10.14±2.85 months. About 18.96% of patients were illiterate, 32.23% were matric, 28.44% were intermediate and 20.33% were graduate and post graduate. Hypertension and diabetes were the commonest co-morbid i.e. 81.3% and 73.7%, hyperlipedimia and smoking were 38.2% and 45% respectively. Frequency of Alzheimer’s disease and vascular dementia in the elderly was observed in 3.79% (16/422) and 2.61% (11/422) cases.

**Conclusion:**

A good number of patients, 27 out of 422, in this hospital based study were suffering from Alzheimer’s disease and vascular dementia. Early detection and prompt treatment can reduce the burden of the disease in our population.

## INTRODUCTION

Dementia is a general term that illustrates the cognitive decline in brain function. There are numerous causes for this condition like Alzheimer’s disease, vascular dementia, Parkinson disease, Huntington disease etc. Alzheimer’s disease is the most frequent type of dementia responsible for more than one half of new cases in Western countries.^1^ With increase in life expectancy in different regions of the world, dementia is appearing as a growing public health concern. Alzheimer’s disease is a progressive degenerative neuropathology. It is also increasing and about 25 million people in the world have been affected by dementia till the first decade of new century, most of them suffering from Alzheimer’s disease. The etiological factors other than older age and genetic susceptibility are yet to be explored.^2^

There are some psychosocial factors associated with Alzheimer’s disease; the cause of Alzheimer is multifactorial that work together in many ways in different people. Alzheimer’s was 2.5 times more likely in people with a history of depression.^3^

Numerous studies have identified association between lifestyle and cognitive decline. It is also reported that Education, physical activity, mentally demanding work, higher managerial positions, various leisure and intellectual activities predict a more favorable cognitive status later in life. A rich social network is also linked to reduced risk of cognitive impairment and dementia.^4^

The prevalence for dementia in people over the age of 60 is greater than 5% in western world and two thirds of them are due to Alzheimer’s disease. Vascular dementia is the second most common cause of dementia in people over age 65 years with a prevalence of 2.2% in Japan.^5^ The mini-mental state exam (MMSE) is the most widely used cognitive test for dementia in clinical practice and also widely used for the purpose of research.^6^

Recent literature shows that worldwide more than 35 million people have Alzheimer’s disease that leads to death within 3 to 9 years after diagnosis. The incidence of the disease doubles every five years after 65 years of age but data on centenarian show that Alzheimer’s disease is not necessarily related to aging.^7^

In Pakistan, there is a dearth of literature on various aspects of Dementia. The lack of awareness and limited available research has under estimated the burden of this disease. This study would help to estimate the frequency of Alzheimer’s and vascular dementia to assess the burden of disease in our population, so that strategies can be planned to reduce mortality and improve quality of life through early detection and prompt treatment of this disease.

## METHODS

In this cross sectional study, data was collected by consecutive sampling method. The study was commenced after formal written permission by hospital ethical committee from 1^st^ October 2013 to 31^st^ March 2014. Informed written consent was taken from all patients who fulfilled the inclusion criteria. Patients with symptoms of dementia for more than 6 months duration, and MMSE score <24 were included in this study. Acutely delirious patients due to any systemic illness e.g. chronic kidney or liver disease, drugs, alcohol, hypothyroidism, hyponatremia, hypoglycemia, sepsis, encephalitis, were excluded. Dementia due to neuropsychiatric disease like Parkinson’s disease, Normal pressure hydrocephalus, depression was also excluded. Similarly patients diagnosed with reversible causes of dementia e.g. hypothyroidism or vitamin B12 deficiency were also excluded. Patients who fell in category of dementia were assessed for duration of symptoms. Demographic information regarding age, gender, marital status, and education were recorded on the pre designed Performa. Information regarding any known co-morbid disease like diabetes, hypertension, hyperlipidemia and smoking were also recorded. Patients underwent CT scan of brain. Patients with generalized atrophy of brain on CT were labeled as Alzheimer’s dementia. While patients with ischemic or hemorrhagic stroke on CT scan of brain was labeled as vascular dementia.

Dementia is a multifactorial disease which covers a wide range of symptoms associated with cognitive decline. It affects memory, thinking, language, judgment and behavior. It was diagnosed on the basis of Mini Mental State Examination score (0-30) and score< 24 was labeled as dementia.^8^

Alzheimer’s causes problems with memory, thinking and behavior. Symptoms usually develop slowly and get worse over time. It was diagnosed on basis of MMSE score <24 and CT (computed tomography) scan of brain showing generalized atrophy (enlarged lateral ventricles and widened sulci) of the brain.^8^

Vascular Dementia was also known as Multi-infarct dementia. It was a form of dementia caused by a series of small strokes. It was diagnosed on basis of MMSE score <24, and CT of brain showing Ischemic (hypodense lesions) or hemorrhagic (hyperdense lesions) stroke.^8^

Data was entered and analyzed using software statistical package for social sciences (SPSS v 15.0). Mean and standard deviation were calculated for numerical variables that is age and duration of symptoms. Frequency and percentages were presented for categorical variables like gender, marital status, education, co-morbid disease (diabetes Mellitus, Hypertension, smoking, and Hyperlipidemia), Alzheimer’s and vascular dementia. Effect modifiers like age, duration of symptoms, gender, and co-morbid conditions were handled by stratified analysis technique. Chi square test was applied. Probability less than or equal to 0.05 was taken as significant.

## RESULTS

There were 422 patients with symptoms of dementia for more than six months duration, and MMSE score<24. The mean age ± SD of the patients was 72.58±5.34 years (95%CI: 72.07 to 73.09), similarly average duration of symptoms was 10.14±2.85 months. Out of 422 cases, 232(54.98%) were male and 190(45.02%) were female. Male to female ratio was 1: 1.22. Abut 18.96% patients were illiterate, 32.23% were matric, 28.44% were intermediate and 20.33% were graduate and post graduate. Hypertension and diabetes were the commonest co-morbid i.e 81.3% and 73.7%, hyperlipedimia and smoking was 38.2% and 45% respectively.

Frequency of Alzheimer’s disease and vascular dementia in the elderly was observed in 3.79% (16/422) and 2.61% (11/422) patinets respectively. According to age groups, frequency of Alzheimer’s disease and vascular dementia was significantly high in 81 to 85 years of age patients while insignificant difference was observed between male and female.

Frequency of Alzheimer’s disease and vascular dementia was significantly associated with hypertension, diabetic mellitus. With respect to duration of symptoms, frequency of Alzheimer’s disease and vascular dementia was insignificant with below and equal to 12 month duration disease and above 12 years.

## DISCUSSION

Frequency of Alzheimer’s disease and vascular dementia in the elderly was observed in 3.79% (16/422) and 2.61% (11/422) of elderly patients in this hospital based study. Various studies have showed that the frequency of dementia in people over the age of 60 is greater than 5% in western world and two thirds of them are due to Alzheimer’s disease. Vascular dementia is the second most common cause of dementia in people over age 65 years with a prevalence of 2.2% in Japan.^5^ In the present study according to age groups, frequency of Alzheimer’s disease and vascular dementia was significantly high in patients aged 81 to 85 years. The etiology of the disease and what causes them are disputed^7^, however with increasing age the aggregation of risk factors may be responsible for increased incidence in that age group. Besides that, concurrence of senile dementia which occurs as a part of normal aging process overlaps and masks the presentation of other forms of dementia resulting in delay in diagnosis and increasing prevalence.^8^

Another study included 14960 residents aged over 65 years in 11 sites in seven low-income and middle-income countries (China, India, Cuba, Dominican Republic, Venezuela, Mexico, and Peru)^9^ that the prevalence varied widely, from less than 1% in the least developed countries, such as India and rural Peru, to 6.4% in Cuba. Our study showed a frequency of 3.79 for Alzheimer’s and 2.61 for vascular dementia which is close to either extreme of the findings of the studies of other developing countries. It is also reported that people in the least developed countries were less likely to report cognitive decline and social impairment,^9^ which means that the prevalence of these disorders may be an under estimation. Yet another study showed that the incidence of Alzheimer’s disease for those aged 65 years and older was 7.7 per 1000 person-years in Brazil,^10^ and 3.24 per 1000 person-years in India.^11^ However, the annual incidence of Alzheimer’s disease in the Yoruba was found to be 1.2%, substantially lower than 2.5% in African Americans from Indiana.^12^

**Table-I:** Frequency of Alzheimer’s disease in the elderly for different age groups, gender, comorbidity and duration of symptoms.

*Variables*	*Stratifications*		*Alzheimer’s disease*		*Total*	*P-Value*	*Chi Square*

			*Yes n=16*	*No n=406*			
Age Groups (Years)	65 to 70 Years		6(3.2%)	179 (96.8%)	185	0.011	9.05
	71 to 80 Years		4(2.2%)	178 (97.8%)	182		
	81 to 85 Years		6(10.9%)	49 (89.1%)	55		
Gender	Male		6 (2.6%)	226 (97.4%)	232	0.15	2.05
	Female		10 (5.3%)	180 (94.7%)	190		
Co-morbid	Diabetes Mellitus	Yes	16	295	311	0.015	-
		No	0	111	111		
	Hypertension	Yes	16	327	343	0.05	
		No	0	79	79		
	Hyperlipidemia	Yes	0	161	161	0.07	
		No	16	246	261		
	Smoking	Yes	6	184	170	0.53	
		No	10	222	232		
Duration of Symptoms	≤ 12months		10 (3.1%)	311 (96.9%)	321	0.19	1.68
	>12 months		6 (5.9%)	95 (94.1%)	101		

**Table-II:** Frequency of vascular dementia in the elderly for different age groups, gender, comorbidity and duration of symptoms.

*Variables*	*Stratifications*		*Vascular dementia*		*Total*	*P-Value*	*Chi Square*

			*Yes n=11*	*No n=411*			
Age Groups (Years)	65 to 70 Years		8 (4.3%)	177 (95.7%)	185	0.012	8.77
	71 to 80 Years		0 (0%)	182 (100%)	182		
	81 to 85 Years		3 (5.5%)	52 (94.5%)	55		
Gender	Male		8 (3.4%)	224 (96.6%)	232	0.23	1.44
	Female		3 (1.6%)	187 (98.4%)	190		
Co-morbid	Diabetes Mellitus	Yes	11	300	311	0.045	-
		No	0	111	111		
	Hypertension	Yes	11	332	343	0.017	
		No	0	79	79		
	Hyperlipidemia	Yes	0	161	161	0.08	
		No	11	250	261		
	Smoking	Yes	8	182	190	0.06	
		No	3	229	232		
Duration of symptoms	≤ 12months		10 (3.1%)	311 (96.9%)	321	0.24	1.36
	>12 months		1 (1%)	100 (99%)	101		

Prevalence estimates of Vascular Dementia in developing countries range from 0.6% to 2.1% in those aged over 65 years. It is also reported that one third of 4.5 million Chinese patients with dementia are predicted to have Vascular Dementia.^13^ With the exception of some Latin American and Asian countries,^14^ Vascular Dementia prevalence in developing countries seems to be low. It is also reported that Vascular Dementia might be more common among the Chinese and Malays, whereas Alzheimer’s disease is common in Indians and Eurasians.^15^ Subcortical Vascular Dementia caused by small-artery disease, associated with hypertensive disease, seems to be a common (73%) cause of Vascular Dementia.^16^

Previous studies on the prevalence of non-cognitive symptoms across the different stages of dementia severity have reported conflicting findings.^17^,^18^ In general, apathy, agitation, aggression and psychotic symptoms appear to steadily increase as the dementia of Alzheimer’s disease progresses, whereas anxiety and depressive symptoms decline from the mild to the moderate/severe stages.^19^ However, Vascular Dementia, anxiety, depression and psychosis have been shown to steadily increase with the progression of the disease.^20^ All these behavioral and psychiatric disturbances vary according to dementia severity. Studying the non-cognitive disturbances can help in the differential diagnosis of dementia subtypes.^21^ However the constellation of symptoms are consistently present in all studies, including ours, and helps warrant investigation to reach a diagnosis. Dementia prevalence estimates vary widely within developing countries which could be due to age structure, genetics, and lifestyle, but could also be due to difficulty in standardizing dementia assessment and reduced survival after diagnosis.^22^

Our study helps to determine the frequency of a disease such as dementia where very little studies have been performed. There is dearth of local data despite the fact that the increasing prevalence of dementia is projected in the sub-continent.^23^

### Limitation of the study

It is a single center hospital based study, hence it cannot represent the frequency or prevalence in general population at large as such. It also lacks control group. Multicenter and double blind studies are required to further assess the risk stratification and to diagnose dementia in our community. Furthermore CT alone may not be enough to diagnose vascular dementia.

## CONCLUSION

Frequency of Alzheimer’s disease and vascular dementia is fairly high in our population. Early detection and prompt treatment can reduce the burden of the disease in our population.
